# Modeling sequential context effects in diagnostic interpretation of screening mammograms

**DOI:** 10.1117/1.JMI.5.3.031408

**Published:** 2018-03-19

**Authors:** Folami Alamudun, Paige Paulus, Hong-Jun Yoon, Georgia Tourassi

**Affiliations:** a Oak Ridge National Laboratory, Computational Sciences and Engineering Division, Oak Ridge, Tennessee, United States; b Oak Ridge National Laboratory, Health Data Sciences Institute, Oak Ridge, Tennessee, United States; c University of Tennessee, Department of Mechanical, Aerospace, and Biomedical Engineering, Knoxville, Tennessee, United States

**Keywords:** perceptual behavior, eye tracking, mammography, cognitive bias, context effect

## Abstract

Prior research has shown that physicians’ medical decisions can be influenced by sequential context, particularly in cases where successive stimuli exhibit similar characteristics when analyzing medical images. This type of systematic error is known to psychophysicists as sequential context effect as it indicates that judgments are influenced by features of and decisions about the preceding case in the sequence of examined cases, rather than being based solely on the peculiarities unique to the present case. We determine if radiologists experience some form of context bias, using screening mammography as the use case. To this end, we explore correlations between previous perceptual behavior and diagnostic decisions and current decisions. We hypothesize that a radiologist’s visual search pattern and diagnostic decisions in previous cases are predictive of the radiologist’s current diagnostic decisions. To test our hypothesis, we tasked 10 radiologists of varied experience to conduct blind reviews of 100 four-view screening mammograms. Eye-tracking data and diagnostic decisions were collected from each radiologist under conditions mimicking clinical practice. Perceptual behavior was quantified using the fractal dimension of gaze scanpath, which was computed using the Minkowski–Bouligand box-counting method. To test the effect of previous behavior and decisions, we conducted a multifactor fixed-effects ANOVA. Further, to examine the predictive value of previous perceptual behavior and decisions, we trained and evaluated a predictive model for radiologists’ current diagnostic decisions. ANOVA tests showed that previous visual behavior, characterized by fractal analysis, previous diagnostic decisions, and image characteristics of previous cases are significant predictors of current diagnostic decisions. Additionally, predictive modeling of diagnostic decisions showed an overall improvement in prediction error when the model is trained on additional information about previous perceptual behavior and diagnostic decisions.

## 1 Introduction

In clinical practice, timely and accurate diagnostic decisions carry important implications for subsequent monitoring, treatment, prognosis, and quality of life. Decisions on disease occurrence are primarily based on well-defined criteria but, ultimately, the judgment of the practitioner trained for this purpose and shaped by years of experience plays the decisive role. However, studies suggest that, even with experienced practitioners, medical decision-making may also be influenced by psychological factors including perception, cognition, emotion, and other similar factors.^1^

Humans have an innate ability for detecting patterns when presented with static visual stimuli. In addition, when exposed to sequential or dynamic stimuli, humans have an advanced ability to detect sequential patterns.^2^ While the capabilities for processing visual patterns are historically advantageous for certain adaptations for survival, they can pose problems when performing tasks of an independent but sequential nature. As such, when humans are faced with uncertainty while engaging in predictive or discrimination tasks, which are sequential in nature, there is a tendency for perceived patterns to subconsciously influence or bias human decisions based on previous states within the sequence.^3^^–^^5^

In addition to being sequential in nature, medical decisions involve individual cases whose pathologies are independent of one another, thereby creating additional potential for bias, in which the practitioner may erroneously (subconsciously) assume that future probabilities are affected by current outcomes (such as mentally adjusting the prior probability distribution for a given pathology after the occurrence of a streaky sequence). This phenomenon is described in literature as context effect.^6^^,^^7^

### 1.1 Factors Affecting Diagnostic Accuracy

Several factors influence the occurrence of error in the decision process in medical diagnosis. These errors can affect subsequent treatment and have a significant negative impact on patients. In mammography, the characteristics of a case, such as breast density,^8^^,^^9^ physician experience levels, and metrics describing the visual search process have been associated with the occurrence of diagnostic error during the interpretation of mammograms.^10^^–^^14^ Studies have examined the perceptual and cognitive dimensions in visual diagnosis.

Perceptual errors, i.e., errors where the observer fails to recognize an abnormality in an image during the detection phase, are a primary cause of missed diagnoses in radiology.^15^^,^^16^ These types of errors result from cognitive biases,^17^^–^^19^ faulty visual search,^20^^,^^21^ or a combination of both. Since there are interrelated cognitive contributors to visual errors, understanding the cognitive processes involved during the interpretation of images can shed light on inherent biases that occur and how these impact diagnostic decisions.

### 1.2 Local Sequential Context Bias

Prior research has shown that medical decisions can be influenced by sequential context, particularly in cases where successive stimuli exhibit similar characteristics. Laming^22^ investigated systematic errors in diagnostic decisions in screen cervical smears. Laming reported that experienced pathologists made systematic errors resulting from psychological interactions in successive cases and offered recommendations on prevention. This type of systematic error, peculiar to sequential stimulus with similar characteristics (as is the case in mammography), indicates that judgments are influenced by features of and decisions about the preceding case in the sequence of examined cases, rather than being based solely on the peculiarities unique to the present case. This type of cognitive bias, known to psychophysicists as sequential context effect,^23^^–^^25^ is studied in radiology literature and referred to in some cases as context bias.^4^^,^^5^

### 1.3 Global Sequential Context Bias

In contrast to local sequential context bias, global sequential context bias refers to the ability to detect patterns in sequences, such as prevalence in a specific type of case, and subconsciously adjusting *a priori* probabilities accordingly. In clinical practice, the sequential nature of diagnostic imaging, combined with the ability to detect patterns increases the possibility for biased decisions particularly in borderline cases where the practitioner may be less certain of the diagnostic decision. While previous studies have focused on the occurrence of bias resulting from case prevalence recognized within the case sequence (which may be a test-specific phenomena), fewer have studied the effect of prior diagnostic decisions on subsequent ones.

Sequential biases may occur in combination particularly in borderline cases where the contextual similarities with a previous case and the inference of case prevalence in case sequence combine to influence diagnostic decisions. In this work, we examine the diagnostic decisions of radiologists with varied levels of experience to determine if and to what extent contextual biases occur and if these occurrences can be predicted. To this end, we explore correlations between current and present perceptual behavior, diagnostic decisions, and case characteristics. We hypothesize that radiologist’s prior perceptual behavior and cognitive decisions are predictive of current decisions. To test our hypothesis, we tasked 10 image readers of varied experience to conduct blind reviews of 100 x-ray images. Eye-tracking data and diagnostic decisions were collected from each reader while reviewing four-view mammographic cases under conditions mimicking clinical practice.

## 2 Methods

### 2.1 Data Collection Protocol

A description of methods for this study is provided in detail in a previous work.^10^ Briefly, we selected 100 screen-film mammograms from a corpus of mammographic cases available from the University of South Florida’s Digital Database for Screening Mammography^26^ (DDSM). Each case included the craniocaudal (CC) and mediolateral oblique views (MLO) of both breasts with associated ground truth using BI-RADS™ lexicon.^27^ Fifty percent of the selected cases were malignant, whereas the remaining 50% was evenly split between normal and benign pathologies. The parenchymal density ranged between 1 (fatty) to 4 (dense), according to the BI-RADS™ lexicon.

For this study, each of the 10 participants (three board certified radiologists and seven radiology residents) performed a review of all 100 cases and provided a report on location and corresponding BI-RADS™ rating of any suspicious mass. Reviews were conducted independently, and markings and BI-RADS™ ratings were reported by each reader through a custom-built graphical user interface, which allowed for image manipulation on dual displays as customary in clinical practice. Readers were outfitted with H6 head-mounted eye-tracking device, with a 60-Hz sampling rate, designed with eye-head integration from Applied Science Laboratories (ASL, Bedford, Massachusetts). Through the remote eye-tracking device, readers’ eye-position data were recorded to within 0.5-deg visual accuracy. Institutional review board approval was obtained prior to conducting this study. Human subject recruitment and data collection were performed according to a protocol approved by the Oak Ridge Site-Wide Internal Review Board. All readers reviewed and signed an informed consent form prior to participation.

Cases for each reader were presented in a randomized order. Although readers examined an identical set of cases, ordering resulting from the randomization process differed for each one. In addition, readers were permitted to complete the study in multiple sessions to accommodate personal preferences and scheduling conflicts. Some readers completed the experiment in two or more sessions on the same day, whereas others completed the experiment over two or more days.

### 2.2 Data Processing

Details describing data preprocessing is provided in a previous work.^10^ In summary, eye-position data for each reader was aggregated from mammographic views across two displays and first preprocessed to extract a time ordered sequence of fixations along with other associated measures (including fixation duration, interfixation degree, and pupil size) using EyeNAL analysis software from ASL.^28^ Data provided through EyeNAL software can be further processed to extract more informative features for a deeper analysis.

For our analysis, we used raw gaze data from eye movements to characterize overall visual behavior. Specifically, we characterize the trajectory of the scanpath using a metric known as fractal dimension (FD). The scanpath describes the trajectory of eye movements formed when time-ordered fixations or gaze points are connected. From the scanpath, we computed the FD) using the Minkowski–Bouligand box-counting method^29^ as a measure of complexity in search behavior for each case. Information describing the computation and analysis of this metric can be found in detail in a previous work.^10^

The 10 participating readers were grouped into one of three experience levels according to years of training and practice: new trainee resident (N) for those residents with at most two mammo rotations, advanced trainee resident (A) for those residents with greater than two mammo rotations, and expert radiologist (E) for board-certified mammographers. Reported diagnostic decisions for each case were mapped into one of the three case pathologies (normal, benign, and malignant) based on BI-RADS™ rating. Cases without markings (i.e., no scores were given) were designated normal (N); BI-RADS™ ratings {2 and 3} as benign (B); and BI-RADS™ ratings {4A, 4B, 4C, and 5} as malignant (M). Three breast parenchyma density groupings were formed by combining heterogeneous and dense parenchymal cases in the same density grouping because of a small sample size.

Data for each reader were structured into overlapping pairs such that the n’th case ($C_{n}$) was paired with k previous cases ($C_{n-i}$, where i is an integer between 0 and 5) with each n’th case representing the current visual behavior and diagnostic decision, and the i preceding cases representing previous visual behavior and diagnostic decisions. Each pair represents the reader’s diagnostic decision for the current case ($C_{n}$), and the previous behavior and diagnostic decisions (${⋃}_{i=0}^{5}C_{n-i}$), which may have potentially impacted it.

**Fig. 1:**
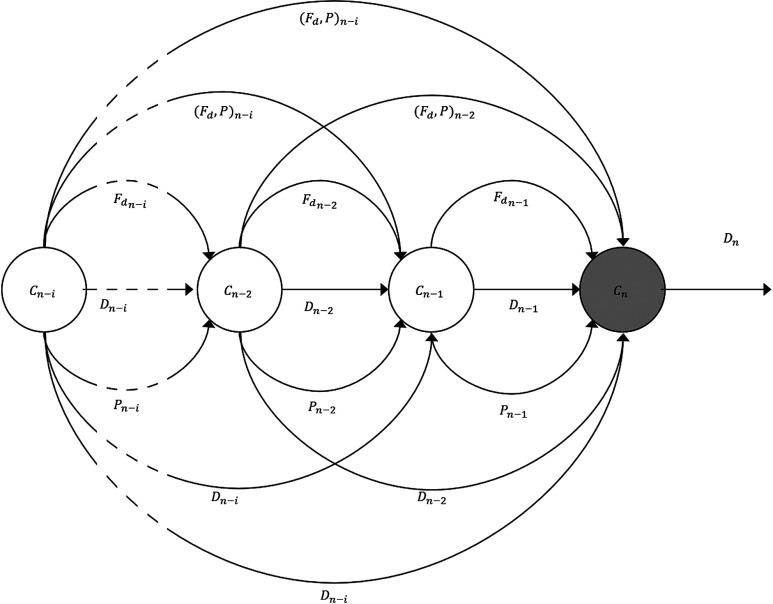
Illustrates potential sequential dependence between diagnostic decisions ($D_{n}$) for a case ($C_{n}$) and the visual behavior (FD $F_{n-i}$), breast parenchymal density ($P_{n-i}$), and diagnostic decisions ($D_{n-i}$) during i previous cases ($C_{n-i}$).

Our analysis was performed on each pair by extracting case descriptors. Namely, visual behavior, computed as the FD of scanpath, diagnostic decisions determined as BI-RADS™ ratings reported by each reader for the respective cases, and image characteristics/properties for which we utilize the breast parenchymal density (Fig. 1).

### 2.3 Statistical Methods

To perform an initial analysis into the temporal relationship in behavior during mammographic screening, we used a statistical tool: autocorrelation function. Autocorrelation captures the Pearson correlation between values of a sequential process at different times, as a function of these times or the lag between them. It enables finding the correlation between previous measures and measures at subsequent time intervals.

In regression analysis using time series data, autocorrelation in a variable of interest is typically modeled with an autoregressive moving average model (ARMA). We represent the sequence of mammographic cases seen by each radiologist as a time series ($C_{1},C_{2},\ldots ,C_{n}$). The autocorrelation function gives the correlation (y axis) between the value of measurements at $C_{n}$ and the value of measurements at $C_{n-k}$ as a function of k, where k is an integer representing the lag (x axis).

To determine any combined or individual dependencies between the current diagnostic decisions and the radiologists’ current and previous visual behavior, previous diagnostic decisions, and characteristics of the current and previous images, we performed a multifactor fixed-effects ANOVA with six levels for diagnostic decision (based on the BI-RADS™ rating provided), three levels for breast parenchyma density, and the FD of scanpaths for eye movements.

## 3 Results

Data from all 10 radiologists who participated in reading mammographic cases were included in this analysis. Each radiologist interpreted 100 cases, resulting in 1000 individual interpretations. We performed analysis on data from each radiologist independently.

### 3.1 Autocorrelation Tests for Randomness

**Fig. 2:**
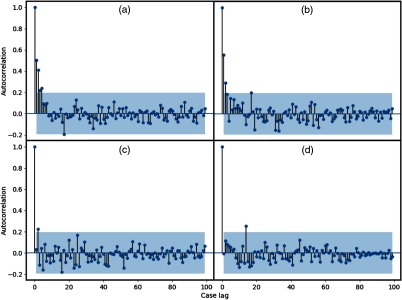
Illustrates autocorrelation function plots for some readers: (a, b) FD of scanpath and (c, d) diagnostic decision.

Autocorrelation is the correlation between a time series lagged one or more time periods and itself. The autocorrelation plot provides a visual representation of the degree of dependence (or independence) between a given time series or sequence and a lagged version of itself over successive intervals. In Fig. 2, we illustrate the autocorrelation function graphs of the FD of scanpaths and of the diagnostic decisions for a subset of readers.

The autocorrelation coefficient summarizes the strength of the relationship between present and past values. The shaded region in the graph represents a 95% confidence interval for significance (i.e., those points within this shaded region do not meet this criteria for statistical significance). From Fig. 2, we note a number of nonsignificant correlation peaks, which tend to indicate a random average distribution. However, in a number of cases we identify several significant (p<0.05) peaks, which show both strong ($r_{k}\geq 0.5$) and weak ($0.5>r_{k}>0.2$) correlation between sequences at their respective time lags.

### 3.2 Analysis of Variance

For our analysis, we restricted previous behavior and diagnostic decisions to five preceding cases. Therefore, each pair of observations consisted of the current diagnostic decision (dependent response variable) and a set of the current visual behavior and breast parenchymal density, along with the diagnostic decision, visual behavior, and breast parenchymal density of the five preceding cases (factors).

A multifactor fixed-effect ANOVA was performed to determine any individual or combined dependencies of the current diagnostic decision on current and previous behavior and diagnostic decisions. The ANOVA was performed individually for each reader. First, we mapped the diagnostic decision for each case to one of seven categories based on the provided BI-RADS™ rating: cases without markings (i.e., no scores or marks were given); BI-RADS™ ratings {2 and 3}; and BI-RADS™ ratings {4A, 4B, 4C, and 5}. We formed three breast parenchymal density groupings (fatty, fibroglandular, and heterogeneous/dense). This was achieved by combining heterogeneous and dense cases in a single group, which became necessary resulting from a small sample size.

Next, we formed pairs of observations with the current diagnostic decision at case n ($D_{n}$) as the response variable, and the current and previous estimates for the FD of scanpath ($F_{n},F_{n-1},F_{n-2},\ldots ,F_{n-5}$) (continuous factor), the parenchymal density for the current and previous cases ($P_{n},P_{n-1},P_{n-2},\ldots ,P_{n-5}$), and previous diagnostic decisions ($D_{n-1},D_{n-2},\ldots ,D_{n-5}$), as independent factors. In Table 1, we summarize the ANOVA results by presenting a subset of statistically significant individual and combined effects.

**Table 1:** Multifactor fixed-effect ANOVA test results with FD (F) of scanpath, prior diagnostic decision (D), and breast parenchymal density (P) for readers by experience level: new (N) and advanced (A) radiology resident, and expert (E) radiologists.

Reader	Factor	F	p>F
E3	$F_{0}:P_{0}:F_{1}:D_{1}:F_{2}:P_{2}:D_{2}$	13.76	$<1e^{-3}$
N1, N2, A3, E1	$F_{0}:F_{1}:F_{3}:F_{4}:F_{5}$	9.17	$<1e^{-3}$
A3, E1	$F_{0}:F_{2}:F_{3}:F_{4}$	8.39	0.01
A3	$F_{0}:P_{0}:F_{1}:P_{1}:D_{1}:D_{2}$	6.97	0.01
N1, N2, A3, E1	$F_{0}:F_{1}:F_{2}:F_{3}:F_{4}:F_{5}$	6.83	0.01
A2, A3	$F_{0}:F_{3}:F_{4}:F_{5}$	7.55	0.01
N1, E2	$P_{0}:F_{0}:F_{1}:P_{1}:D_{1}:F_{2}:P_{2}:D_{2}:F_{3}:P_{3}:D_{3}$	6.40	0.01
E1	$P_{0}:F_{0}:F_{1}:P_{1}:D_{1}:P_{2}:D_{2}$	6.35	0.01
E3	$P_{0}:F_{1}:P_{1}:D_{1}$	6.34	0.01
A2, E2	$F_{0}:F_{1}:P_{1}:D_{1}:F_{2}:P_{2}:D_{2}$	6.20	0.02
N3, A1	$P_{0}:F_{0}:F_{1}:P_{1}:D_{1}:F_{2}:P_{2}:D_{2}:P_{3}:D_{3}$	6.08	0.02
A4	$D_{1}:D_{2}:D_{3}:D_{4}$	6.02	0.02
N1, N2, A4, E3	$P_{0}:F_{0}:F_{1}:P_{1}:D_{1}$	5.94	0.02
A2, E3	$F_{0}:D_{1}:F_{2}:P_{2}:D_{2}$	5.88	0.02
A4	$F_{0}:P_{1}:D_{1}:F_{2}:P_{2}:D_{2}$	5.42	0.02
A2	$P_{0}:F_{0}:F_{1}:P_{1}:D_{1}:F_{2}:P_{2}:D_{2}:P_{3}$	5.33	0.02
N3, A1, A2	$F_{0}:F_{1}:F_{3}:F_{4}$	5.63	0.03
N1	$P_{0}:F_{0}:F_{1}:P_{1}:D_{1}:F_{2}:P_{2}:D_{2}:F_{3}:P_{3}:D_{3}:F_{4}:P_{4}:D_{4}$	5.18	0.03
E2	$D_{1}:D_{2}:D_{3}:D_{5}$	5.21	0.03
N1, E1, A1, A3	$P_{0}:F_{0}:F_{1}:P_{1}:D_{1}:F_{2}:P_{2}:D_{2}:F_{3}:P_{3}:D_{3}:F_{4}:P_{4}:D_{4}:P_{5}:F_{5}$	5.11	0.03
E2	$P_{0}:F_{0}:F_{1}:P_{1}:D_{1}:F_{2}:P_{2}:D_{2}:F_{3}:D_{3}$	4.36	0.04
N1	$F_{1}:P_{1}:D_{1}:F_{2}:P_{2}:D_{2}$	4.37	0.04
N1, A4	$P_{0}:F_{1}:D_{1}:F_{2}:P_{2}:D_{2}$	4.21	0.04

### 3.3 Predictive-Model-Based Analysis

As described in Sec. 3.1, each data point consists of a pair: diagnostic decision ($D_{n}$) at case $C_{n}$ as target, and reader’s diagnostic decision and behavior for $C_{n-i}$ previous cases, where 0≤i≤5 (our analysis was limited to a maximum of five previous cases) as features. This process results in six distinct datasets representing ${⋃}_{i=0}^{5}(C_{n},C_{n-i})$ pairings with a maximum of m=100−i samples for each subject in each dataset. During the experimental protocol (described in Sec. 2.1), readers were permitted to complete the experiment over multiple sessions. The final number of pairs for each reader varied 85 to 98 (with a median value of 94) dependent upon the number of sessions, resulting in m=100−i×(s+1) pairs, where s is the total number of sessions.

To assess the predictive potential of the observed dependencies between current diagnostic decisions and previous behavior, we developed a predictive model using a random forest classification model. The model was evaluated using a within-subject leave-one-case-out cross-validation model evaluation scheme. To achieve this, data were first partitioned into 10 independent datasets, representing response data from each of the 10 readers. Leave-one-case-out cross-validation scheme partitions each dataset into complementary subsets, where all but a single data sample is utilized in model-training (training set), which analysis is validated on the excluded sample (test case). This process is repeated until every sample in the dataset is utilized as the singular test case, resulting in m models (where m is the total number of samples).

The aggregated predictive values over all rounds serve as the final performance evaluation of the predictive model for each user. In this paper, we report the weighted-average f-score (the harmonic mean of precision and recall) performance metrics for predicting diagnostic decision on a current case using features derived from previous behavior and diagnostic decisions. In Table 2, we report the f-score for each user by i (number of previous cases) and, for comparison purposes, Table 2 also includes performance metrics for a random chance classifier (a random classifier provides a simple baseline reflecting the predictive outcome of a random guess).

**Table 2:** Performance (f-score) of behavior-based prediction task for readers by experience: new (N) and advanced (A) radiology resident and expert (E) radiologists.

Reader	$C_{n}$	$C_{n-1}$	$C_{n-2}$	$C_{n-3}$	$C_{n-4}$	$C_{n-5}$	Random chance
N1	0.39	0.47	0.42	0.50	0.53	0.49	0.24
N2	0.85	0.90	0.87	0.85	0.89	0.87	0.27
N3	0.61	0.67	0.68	0.63	0.60	0.61	0.37
**Avg.**	**0.62**	**0.68**	**0.66**	**0.66**	**0.67**	**0.66**	**0.29**
A1	0.30	0.39	0.29	0.31	0.34	0.40	0.29
A2	0.55	0.47	0.50	0.53	0.46	0.50	0.33
A3	0.57	0.59	0.63	0.63	0.68	0.67	0.33
A4	0.51	0.43	0.42	0.44	0.42	0.42	0.38
**Avg.**	**0.48**	**0.47**	**0.46**	**0.48**	**0.48**	**0.50**	**0.33**
E1	0.51	0.57	0.53	0.49	0.52	0.48	0.29
E2	0.41	0.43	**0.46**	0.52	0.45	0.48	0.44
E3	0.38	0.51	0.54	0.43	0.50	0.53	0.28
**Avg.**	**0.43**	**0.50**	**0.51**	**0.48**	**0.49**	**0.50**	**0.34**

## 4 Discussions and Conclusion

In this study, we examined the potential for bias in diagnostic by analyzing visual behavior and diagnostic decisions of 10 radiologists viewing mammograms for breast cancer screening. To this end, we performed autocorrelation tests, analyses of variance, and predictive-model-based analysis on the diagnostic decision and visual behavior (characterized using the FD of scanpath) for individual readers’ case sequences to determine if temporal dependencies exist between current and previous behavior and decisions.

The results from the autocorrelation tests show significant (p<0.05), strong ($r_{k}\geq 0.5$), and weak ($0.5>r_{k}>0.2$) correlation $r_{k}$, in sequences of both visual behavior and diagnostic decisions for 7 of the 10 subjects reported in this study. We also note the occurrence of stronger sequential dependencies, indicated by large correlation coefficient ($r_{k}$) in visual behavior in comparison to diagnostic decisions, which show a weaker correlation.

The ANOVA tests conducted determine a number of significant individual and combined effects on current diagnostic decisions. These results, presented in Table 2, suggest that visual search behavior during previous cases can serve as combined predictors of current diagnostic decision. In addition, visual search behavior, breast parenchymal density, and diagnostic decisions during previous cases are combined predictors of current diagnostic decisions. This observation is consistent across readers with a variation in the combination of factors.

The predictive-model-based analysis was performed on a random forest classification model trained using a leave-one-out cross-validation scheme on each individual reader. Our results show an f-score of 0.54 averaged over all case-pairing datasets, which represents a 64% improvement over random chance (0.33). The notably higher predictability of the current diagnostic decision of new radiology residents (0.66 f-score averaged over all case-pairing datasets) using data from previous visual behavior and diagnostic decisions, compared with advanced radiology residents (0.48 f-score averaged over all case-pairing datasets), and experienced radiologists (0.49 f-score averaged over all case-pairing datasets), indicates that new residents may be more susceptible to sequential context bias. However, data from the current experiments are not sufficient to establish the significance of this observation. The predictive performance results also show, with the exception of two readers (both advanced radiology residents), an increase in performance when information from previous cases is introduced into the model [$M_{n}<{⋃}_{i=0}^{5}(M_{n},M_{n-i})]$).

This study represents a step in the investigation of contextual biases in medical diagnosis through the incorporation of sensor-based measurements of visual behavior in the analysis of biases in mammographic readings. Our findings suggest that new radiology residents are most susceptible to context bias, causing their diagnostic decisions to be predictable given previous behavior and diagnostic decisions. However, our findings also suggest that more advanced radiologists experience some level of context bias.

There are limitations of this study, which need to be considered. The first and foremost is the inherent nature of experiments of this nature. The results from this test indicating contextual bias may be an artifact of the experimental design. We are unable to determine whether bias observed in participating radiologists reflects their individual altered expectations of case prevalence within the study or, if it is applicable, in clinical practice.

Further, interruptions during multiple sessions in all participating readers resulted in the exclusion of ∼15% of data collected in this study. Our analysis treats these discontinuities as the beginning of a new experiment by ignoring the first few samples, the number of which is dependent on the number of previous cases being considered. However, it is possible that a number of sessions were interrupted for the purpose of clinical diagnosis as experiments were performed in the clinician’s work place. Our analyses do not factor the influence of these intermediary cases on diagnostic decisions after the reader resumes the experiment.

In conclusion, breast parenchymal density, visual behavior, and diagnostic decisions in previous cases may serve as predictors of current diagnostic decisions indicating contextual bias in radiologists’ review and diagnosis of mammographic images in testing situations. We are unable to ascertain the veracity of these claims in clinical practice. However, in the event that these observations apply in clinical practice, a deeper understanding of how these biases occur, and additional factors, which improve predictability of these biases, will be invaluable in improving training methodology and reducing the occurrence of errors in diagnostic imaging.
